# Reproducibility of published model-based cancer drug cost-effectiveness analyses: a study protocol for a cross-sectional analysis

**DOI:** 10.1136/bmjopen-2024-096719

**Published:** 2025-06-20

**Authors:** Mikael Svensson, Jonathan Siverskog, Naimi Johansson, Martin Henriksson

**Affiliations:** 1 University of Gothenburg, Gothenburg, Sweden; 2 Department of Economics, Linköping University, Linköping, Sweden; 3 Department of Clinical Science and Education, Karolinska Institute, Stockholm, Sweden; 4 Department of Health, Medicine and Caring Sciences, Linköping University, Linköping, Sweden

**Keywords:** Health Care Costs, HEALTH ECONOMICS, STATISTICS & RESEARCH METHODS

## Abstract

**Abstract:**

**Introduction:**

Model-based cost-effectiveness analysis (CEA) of pharmaceuticals informs reimbursement and pricing in many healthcare systems, and it is essential that CEA evidence is valid and reliable. Several studies have reported lacking transparency in CEA studies. In this study protocol, we describe a study that will investigate whether model-based CEA studies of cancer drugs are transparent and informative enough to enable the reproduction of study findings.

**Methods and analysis:**

This study protocol outlines a study where we will identify CEA studies indexed in MEDLINE from 2015 to 2023 based on predefined search terms. We will include English-language CEA studies evaluating pharmaceutical treatments based on decision-analytical modelling methods that report cost-effectiveness results using life-years, quality-adjusted life-years and/or disability-adjusted life-years as health outcome metric(s). Two authors will screen abstracts and full text for inclusion. We anticipate that a maximum of 150 studies will be included after a full-text review. A data extraction template is designed to capture information used to determine reproducibility together with other information that will be analysed as potential determinants of reproducibility in logistic and linear regression analyses.

**Ethics and dissemination:**

This study design has been deemed exempt from ethical approval. All collected data will be made available in an online repository that will host the study protocol and other supplementary data. Results from this proposed study will be published in peer-reviewed journals and at scientific conferences and workshops.

**STRENGTHS AND LIMITATIONS OF THIS STUDY**

- To our knowledge, the first study to assess reproducibility of model-based cost-effectiveness analyses of cancer drugs.
- A comprehensive search strategy enhances the likelihood of identifying relevant studies with a good representation of the field of model-based cost-effectiveness studies of cancer drugs.
- A limitation is that we assess reproducibility based on a set of predefined criteria as opposed to actually attempting to reproduce each and every identified study.

## Introduction

 Economic evaluations of pharmaceutical treatments—the formal modelling and comparison of benefits, harms and costs of a new drug—play an increasingly important role in priority setting such as reimbursement decisions and guideline development in many healthcare systems around the world.^1^,^3^ Economic evaluation of pharmaceuticals is typically carried out using cost-effectiveness analysis (CEA), where the incremental cost of a treatment is related to the incremental health effects, measured in, for example, life-years (LYs) or quality-adjusted life-years (QALYs) gained. Considering the policy and population health impacts that can result from the body of CEA evidence, it is essential that the evidence is valid and reliable.

There is an increasing discussion within science regarding how much research findings really can be trusted. In a seminal paper, Ioannidis argued that it is more likely for an empirical research claim to be false than true.^4^ More recently, there has been a growth of studies that try to assess the replicability and reproducibility of research findings. Replicability refers to whether results can be repeated with new data. In contrast, reproducibility refers to whether results can be reproduced using the same data and analysis as in the original study.^5^,^10^ It could be argued that cost-effectiveness research can be particularly vulnerable to replicability and reproducibility issues, considering that in decision models (eg, state-transition models), there are many complex trade-offs when specifying the model structure and parameter assumptions^11^ that imply that the researcher’s df may be more prominent than in many other fields.^12^,^14^ There is also the added dimension that CEA findings play an important part in market access and reimbursement decisions, implying that financial incentives may lead to skewed assumptions favouring a particular treatment.^15^

Studies that have assessed replicability in experimental psychological and social science studies have found that around 50% of results are replicable.^16 17^ Rates of reproducibility have also been low in the economics and social science literature.^5 18 19^ For example, in a study trying to reproduce the results of 59 papers in well-regarded economics journals, only one-third were reproducible based on data, code and information available in the studies, and the reproducibility increased to no more than 49% after the authors of the original studies were consulted to assist the reproducibility.^5^ Recently, a few studies have assessed the transparency, openness and potential reproducibility of CEA decision models. In one study, a number of research teams tried to reproduce the results from two diabetes models and ended up with substantial variation in results across teams, and no team was able to reproduce the original study findings.^20^ In a study by Catalá-López *et al*,^21^ which resembles the study outlined in this study protocol, the authors reviewed 200 published CEA studies to assess if enough information was presented in each study to facilitate reproducibility. They reported that up to 56% of studies contained enough information to be (theoretically) reproducible.

We aim to build on this literature to assess the reproducibility of CEA decision models, with a specific focus on cancer drug treatments. We concentrate on cancer drugs since a large share of approved drugs in the last 10–15 years have been in the oncology space, and it is a field where there have been considerable discussions around the (increasing) costs with a substantial economic impact on healthcare systems.^22^,^24^ It should be noted that reproducibility is a necessary, but not a sufficient criterion for a CEA decision model to provide meaningful input to decision-making. It also requires model validation, defined as ‘the act of evaluating whether a model is a proper and sufficient representation of the system it is intended to represent’.^25^ In principle, a CEA decision model can be fully reproducible but still provide invalid input to decision-making because it does not, for example, characterise the disease process correctly. However, reproducible reporting is a vital first step to check the trustworthiness of CEA decision models. Therefore, this protocol outlines a study that aims to investigate the reproducibility of CEAs of cancer drugs based on the transparency in the reporting of model details. The protocol also outlines analyses to estimate the relationship between reproducibility and study characteristics.

## Methods and analysis

Reproducibility has been defined in terms of computational, recreate and robustness reproducibility.^6^ Computational reproducibility is when data and code are available in the original study to enable reproduction. Recreate reproducibility refers to whether an original study contains enough information and assumptions such that external parties can reproduce results even though the specific code or model is not available. Finally, robustness reproducibility refers to the degree to which the original study results are robust to different, but still plausible, modelling choices of the same data and assumptions. In this project, we will assess if published studies report enough information to be computationally reproducible or recreate reproducible.

### Study inclusion and exclusion criteria

The selection of studies will be based on the PICO framework,^26^ and we will consider studies that adhere to the following:

- Population (P): any cancer population.
- Intervention (I): any pharmacological treatment that is an intended curative or non-curative treatment of the cancer illness.
- Comparator (C): standard of care, another active treatment (ie, not necessarily standard of care) or placebo.
- Outcome (O): the incremental cost-effectiveness ratio expressed as the cost per LY, QALY or averted disability-adjusted life-year.

In terms of study designs, we include full cost-effectiveness studies that rely on decision-analytical modelling (eg, decision-trees, Markov and semi-Markov models, discrete event and agent-based models). We include studies from any country or healthcare context published between 2015 and 2023.

The exclusion criteria are:

- Studies lacking an abstract and/or full-text version in English language.
- Studies where the intervention drug targets a toxicity or side effect of a cancer treatment rather than the cancer itself.
- Studies where the pharmacological treatment comes as a secondary consequence of an evaluated screening or diagnostic test (eg, the model evaluates some diagnostic test used to guide subsequent pharmacological treatments).
- Studies solely relying on within-trial data analysis.
- Publications in the forms of editorials, letters, commentaries, perspectives/discussion articles and analyses published only as conference abstracts.

### Search and screening strategy

Based on the PICO, search terms were developed with a library information specialist (table 1) and the MEDLINE/PubMed database was searched, resulting in 696 abstracts. Two authors will independently screen all abstracts using the Covidence systematic review software. Any assessment discrepancies between reviewers will be resolved by consensus or involving a third author. Full-text papers will be read by two authors to decide on final inclusion based on satisfying the PICO, and again, any discrepancies will be resolved by consensus or involving a third author.

**Table 1:** Search terms

Block	Search words
#1 Pharmaceutical treatments	(Drug Therapy(Majr) OR “drug therapy”(Subheading))
#2 Cancer disease	(“Neoplasms”(Mesh))
#3 CEA/CUA studies	(“Costs and Cost Analysis”(Majr:NoExp)) OR “Cost-Effectiveness Analysis”(Mesh)) OR “Cost-Benefit Analysis”(Majr)) OR “Drug Costs”(Majr))
#4 Time restriction	
#5 Study design restriction	Editorial(pt) OR Letter(pt) OR Historical Article(pt) OR Meta-Analysis(pt) OR Retracted Publication(sb) OR Review(pt) OR systematic(sb)

Note: search based on: #1 AND #2 AND #3 AND #4 NOT #5.

CEA, cost-effectiveness analysis; CUA, cost-utility analysis; MESH, Medical Subject Headings.

### Sample size

**Figure 1:**
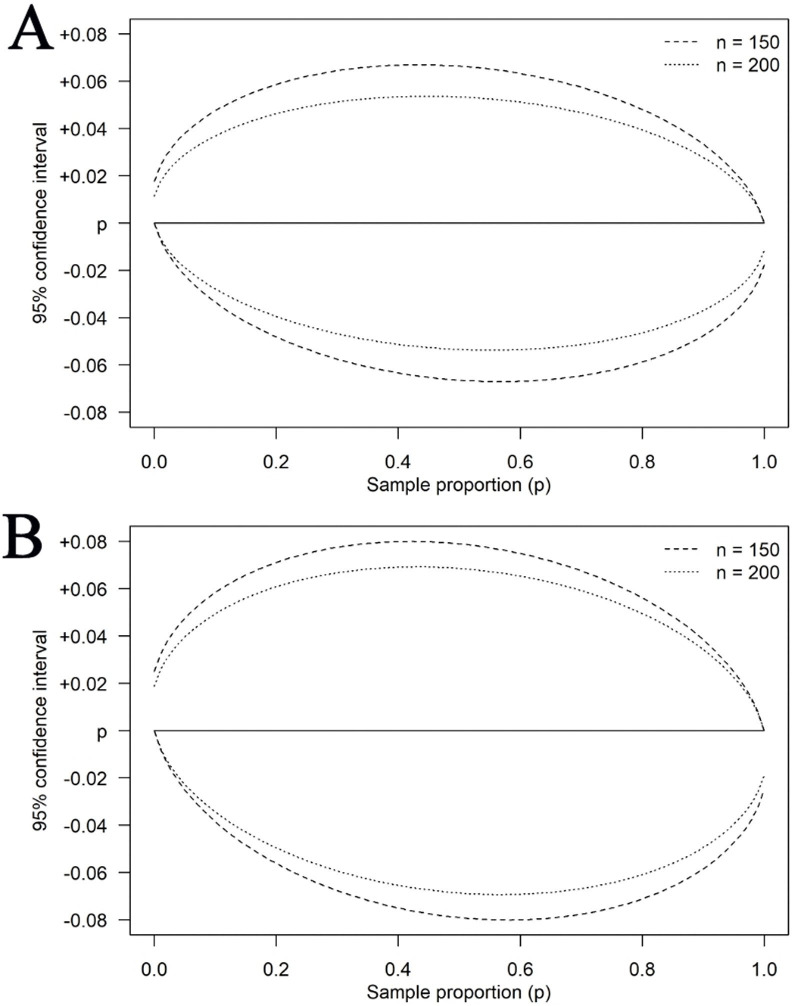
Width of a 95% CI (Wilson score method) for a population proportion (eg, share of recreate reproducible studies) with our selected sample size (n=150) and a moderately larger sample (n=200) over the range of potential sample proportions; (A) finite population, N=500; (B) infinite population.

If the population of studies that meet the inclusion criteria after full-text review is larger than 150, a random sample of 150 papers will be drawn for data extraction. This number was selected based on feasibility given the resource and time constraints of the project. Because the main objective of our study is descriptive and will assess several indicators, a power calculation was not deemed relevant. Still, an analysis was undertaken to ensure that n=150 would not result in CIs so wide as to be uninformative about population proportions and that a moderate increase in sample size would not lead to a gain in precision large enough that it was deemed motivated with an increase (figure 1). If the number of studies meeting the inclusion criteria does not reach 150, data will be extracted from all of the included studies (ie, the analysis includes the full population of studies).

### Data extraction

The data extraction template was created with insight from several of the available checklists^27^ and a recent study by Catalá-López *et al*.^21 28^ Since we deemed no existing checklist/template to fully satisfy our aim to evaluate reproducibility through transparency and use of open reporting, we have designed a new bespoke checklist for our purpose. The checklist was designed in a stepwise procedure with pilot testing on published studies to iteratively implement relevant improvements and clarifications. We will use the checklist to extract data from the included studies, with two authors independently extracting data from each study. Any conflicts in the extracted data will be resolved by consensus. The full data extraction template is shown in table 2.

**Table 2:** Data extraction template

Item #	Part A – study characteristics
	**General paper and journal characteristics**
1	DOI
2	Paper title
3	Year of publication
4	First author
5	Which journal is the health economic evaluation published in?
6	Is the journal a clinical or a health economics journal?
7	Number of authors
	**Disease context and healthcare setting**
8	What is the cancer indication?
9	What is the base case population analysed in the health economic evaluation?
10	For which country is the analysis carried out?
11	What was the intervention treatment(s)?
12	What was the control arm treatment(s)?
13	Which health outcome measures were used? If several, code all that were used.
	**Model perspective and policy parameters**
14	What type of health economic study design and model was used?
15	What was the perspective of the health economic evaluation?
16	What time horizon was used? (years)
17	Which discount rate was used for costs?
18	Which discount rate was used for health outcomes?
	**Funding and conflicts**
19	What was the funding source?
20	Was conflict of interest reported?
21	Open comment
	**Pre-registration and software**
22	Was a pre-registered study protocol/health economic analysis plan used and referenced?
23	Which software(s) was used to conduct the study?
	**Part B – reproducibility and transparency checklist**
	**Computational reproducibility**
24	Was the code or spreadsheet model openly available?
	**Model structure**
25	Were all health states and/or events of the decision model completely described?
26	Were all possible transitions/pathways between states and/or events completely described?
	**Data inputs: transitions**
27	For state transition models, were all transition probabilities provided?
28	For partitioned survival/discrete event models, was the specific parametric survival model described including results on coefficient estimates and/or all curves available to digitise?
	**Data inputs: costs**
29	Were all cost inputs available to reproduce costs with each treatment/state/event?
	**Data inputs: health outcomes**
30	If relevant (QALY/DALY outcomes), were all HRQoL/disability weight inputs available to reproduce outcomes with each treatment/state/event?
	**Sensitivity analysis reporting**
31	If PSA was used, were statistical distributions and ranges/parameters described?
32	If DSA was used, were the ranges described?
	**Results reporting**
33	Was the total cost and health outcomes reported for each treatment arm separately in the base-case?
34	Was the base-case result reported as an ICER?
35	Was the base-case result reported as a net benefit measure?
	**Part C – summary measures**
	**Estimated reproducibility and degree of transparency**
36	Was enough information reported for the health economic evaluation to be computationally reproducible?
37	Was enough information reported for the health economic evaluation to be recreate reproducible?
38	Degree of transparency (score 0–4)

DALY, disability-adjusted life-year; DOI, digital object identifier; DSA, deterministic sensitivity analysis; HRQoL, health-related quality of life; ICER, incremental cost-effectiveness ratio; PSA, probabilistic sensitivity analysis; QALY, quality-adjusted life-years.

The data extraction template contains a total of 38 items, initially covering general paper and journal characteristics (items 1–7), the disease context and healthcare setting (items 8–13), basic model perspective and policy parameters (items 14–18), funding and conflicts of interests (items 19–21), pre-registration and software (items 22–23). The second part of the template focuses on reproducibility and transparent reporting (items 24–35). The three final items are the reproducibility outcome scores (items 36–38), which are automatically populated based on responses to items 24–35.

### Outcomes

The assessment of reproducibility will be summarised and analysed using three reproducibility outcome measures (see table 3 for details on coding):

- Computationally reproducible: dichotomous variable (yes/no) if the study contains the model and/or code in paper or supplement such that an external researcher can reproduce the results.
- Recreate reproducible: dichotomous variable (yes/no) if the study is deemed to contain enough information such that an external researcher can reproduce results even though code or model is not available. In short, information will be deemed insufficient unless a study reports all transition probabilities (for state-transition models) or parameter estimates (for survival models), and all other inputs required to calculate total costs and health outcomes with each treatment.
- Degree of transparency: the sum of scores from items 25–30 will be used to differentiate studies where, for example, one piece of information is lacking for reproducibility from studies missing almost all information. As reproducibility is a binary concept, we refer to this outcome as the degree of transparency.

**Table 3:** Outcome variables coding

Outcome variable	Coding
Recreate reproducible	Yes (1) if fulfilling items 25 AND 26 AND 27 OR 28 AND 29 AND 30, No (0) otherwise
Computationally reproducible	Yes (1) if fulfilling item #24 (Table 2), No (0) otherwise
Degree of transparency	Scored between 0 and 4 (4 highest degree of transparency) based on:If yes items #25 AND #26=1; If yes items #27 OR #28=1; If yes item #29=1; If yes item #30=1.

### Statistical analysis

The three outcome variables will be summarised descriptively using summary statistics and CIs for the proportion or mean in the sample of included studies. Further, we will analyse potential predictors of the three outcome variables using logistic, linear and ordinal regression models. The predictors that we will include to assess potential associations with reproducibility are journal impact factor, year of publication, type of cancer modelled, patient characteristics, type of pharmacoeconomic model, study perspective, time horizon of evaluation, CEA results, type of funding and presence of study protocol. The predictors will mostly be coded as categorical variables, except for variables that can be considered continuous (journal impact factor, time horizon and modelling results will be coded as continuous variables).

### Registration

We have registered this study protocol with the Open Science Framework (https://osf.io/7zw24/). It is anticipated that the data extraction will be implemented from November 2024 to May 2025, and data analysis will begin in June 2025.

### Patient and public involvement

This study does not include any patients or the public in study design, analysis or interpretation.

## Ethics and dissemination

This study will be based on previously published peer-reviewed papers, and formal ethical review board approval is not a requirement. The dissemination plan includes sharing results and our interpretation of results in open-access peer-reviewed publications, scientific conferences, as well as in relevant social media.

### Amendments

The study protocol will be amended if deemed necessary for a successful implementation of the study. Any such amendments will be reported in the final publication of study findings and transparently published in the OSF repository for this project (https://osf.io/7zw24/).

## Discussion

This study protocol outlines a study to assess the reproducibility of model-based CEAs of cancer-drug treatments. For a study that is fully reproducible, it should be possible to duplicate the study results using the same assumptions and analysis as in the original study. To be able to do this, it is necessary that the reporting is fully transparent and include all the required details to reproduce study findings on costs, health outcomes and cost-effectiveness. As mentioned, reproducible CEA studies do not necessarily imply valid studies, as a poorly designed model can also be made reproducible. Still, it is an essential first check of credibility and trustworthiness. As a field, economic evaluation in healthcare is potentially even more exposed to reproducibility problems given the large number of data and modelling assumptions required by the researcher, implying that transparent and open reporting can be considered especially vital to establish credibility for the field.

We have developed inclusion and exclusion criteria based on the PICO framework, a systematic search strategy focusing on papers published between 2015 and 2023. We decided to focus on cancer-drug treatments, considering that this area has seen rapidly increasing pharmaceutical costs and thus is particularly relevant from a health economics perspective. Since we are not including model-based CEAs in general, the conclusions that can be drawn from the planned study cannot necessarily be generalised to the entire CEA field. Another limitation is that we will assess reproducibility based on the transparency and openness in reporting and will not attempt to actually reproduce study results from the included studies.

The results from the study will be disseminated through open-access publications and scientific conferences, and we believe that the findings from the planned study can provide valuable input to inform improved research practices and reporting so as to increase the credibility of the CEA field.
