# Supplementary material for: Reproducibility of published model-based cancer drug cost-effectiveness analyses: a study protocol for a cross-sectional analysis
Source: BMJ Open. 2025 Jun 20;15(6):e096719. doi: 10.1136/bmjopen-2024-096719 (PMC12182205; doi:10.1136/bmjopen-2024-096719)
Supplement: Supplementary file 1 [file bmjopen-2024-096719.reviewer_comments.pdf]

PEER REVIEW HISTORY

BMJ Open publishes all reviews undertaken for accepted manuscripts. Reviewers are asked to complete a checklist review form and are provided with free text boxes to elaborate on their assessment. These free text comments are reproduced below.

ARTICLE DETAILS

Title (Provisional)

The reproducibility of published model-based cancer drug cost-effectiveness analyses: a study protocol for a cross-sectional analysis

Authors

Svensson, Mikael; Siverskog, Jonathan; Johansson, Naimi; Henriksson, Martin

---

VERSION 1 - REVIEW

**Reviewer**	**1**
**Name**	**Feldman, Inna**
**Affiliation**	**Uppsala Universitet, Department of Public Health and Caring Science**
**Date**	**16-Jan-2025**
**COI**	**None**

This is very interesting project plan with aim to summarize the data and evidence on the reproducibility of published cancer drug cost-effectiveness analyses. The subject is very important and the results of the study will benefit scientific literature.

I have some comments and suggestions that might contribute to the manuscript.

1. As I understood, the authors are manly focused on model-based CEA, but this is not so clear from the title, suggest to name the article as “The reproducibility of published cancer drug model-based cost-effectiveness analyses….”

2. Why to restrict the search to 2023, not to 2024?

3. This is not so clear how the authors will select the only model-based studies, during the screening?

4. Data extraction: Model perspective & policy parameters – suggest to add “type of health economic model”, additionally to other parameters

---

VERSION 1 - AUTHOR RESPONSE

Reviewer #1:

I have some comments and suggestions that might contribute to the manuscript.

1. As I understood, the authors are manly focused on model-based CEA, but this is not so clear from the title, suggest to name the article as “The reproducibility of published cancer drug model-based cost-effectiveness analyses….”

Our response: Good point, and we have revised the title in line with the suggestion.

2. Why to restrict the search to 2023, not to 2024?

Our response: We initiated the plan for this study in the summer/fall of 2024 (protocol submitted fall 2024), and we therefore decided to have an end date for inclusion at the end of the last full year. Although we now realize that the full year of 2024 has passed, we do not think this will influence the findings in a meaningful way.

3. This is not so clear how the authors will select the only model-based studies, during the screening?

*Our response: Yes, we agree that this was unclear. We have added another item in the exclusion criteria now that make this more clear (see page 7, exclusion: “*Studies solely relying on within-trial data analysis”).

4. Data extraction: Model perspective & policy parameters – suggest to add “type of health economic model”, additionally to other parameters

Our response: This was actually part of item #14 in our plan, but we realize that was not made clear from the description. We have now added “type of model” to this item..
